# Perceptions of European ME/CFS Experts Concerning Knowledge and Understanding of ME/CFS among Primary Care Physicians in Europe: A Report from the European ME/CFS Research Network (EUROMENE)

**DOI:** 10.3390/medicina57030208

**Published:** 2021-02-26

**Authors:** John Cullinan, Derek F. H. Pheby, Diana Araja, Uldis Berkis, Elenka Brenna, Jean-Dominique de Korwin, Lara Gitto, Dyfrig A. Hughes, Rachael M. Hunter, Dominic Trepel, Xia Wang-Steverding

**Affiliations:** 1 School of Business & Economics, National University of Ireland Galway, University Road, H91 TK33 Galway, Ireland; 2 Society and Health, Buckinghamshire New University, High Wycombe HP11 2JZ, UK; derekpheby@btinternet.com; 3 Department of Dosage Form Technology, Faculty of Pharmacy, Institute of Microbiology and Virology, Riga Stradins University, Dzirciema Street 16, LV-1007 Riga, Latvia; diana.araja@rsu.lv (D.A.); Uldis.Berkis@rsu.lv (U.B.); 4 Department of Economics and Finance, Università Cattolica del Sacro Cuore, Largo Agostino Gemelli 1, 20123 Milan, Italy; elenka.brenna@unicatt.it; 5 Internal Medicine Department, University of Lorraine, 34, Cours Léopold, CS 25233, 54052 Nancy CEDEX, France; jean-dominique.dekorwin@univ-lorraine.fr; 6 University Hospital of Nancy, Rue du Morvan, 54511 Vandoeuvre-Lès-Nancy CEDEX, France; 7 Department of Economics, University of Messina, Piazza Pugliatti 1, 98122 Messina, Italy; lara.gitto@unime.it; 8 Centre for Health Economics & Medicines Evaluation, Bangor University, Bangor LL57 2PZ, UK; d.a.hughes@bangor.ac.uk; 9 Research Department of Primary Care and Population Health, Royal Free Medical School, University College London, London NW3 2PF, UK; r.hunter@ucl.ac.uk; 10 School of Medicine, Trinity College Dublin, College Green, D02 PN40 Dublin, Ireland; trepeld@tcd.ie; 11 Global Brain Health Institute, Trinity College Dublin, D02 PN40 Dublin, Ireland; 12 Global Brain Health Institute, University of Califonia, San Francisco, CA 94143, USA; 13 Warwick Medical School and Zeeman Institute, University of Warwick, Coventry CV4 7AL, UK; xiasteverding@gmail.com

**Keywords:** ME/CFS, myalgic encephalomyelitis, chronic fatigue syndrome, primary care, GP knowledge and understanding

## Abstract

*Background and Objectives*: We have conducted a survey of academic and clinical experts who are participants in the European ME/CFS Research Network (EUROMENE) to elicit perceptions of general practitioner (GP) knowledge and understanding of myalgic encephalomyelitis/chronic fatigue syndrome (ME/CFS) and suggestions as to how this could be improved. *Materials and Methods:* A questionnaire was sent to all national representatives and members of the EUROMENE Core Group and Management Committee. Survey responses were collated and then summarized based on the numbers and percentages of respondents selecting each response option, while weighted average responses were calculated for questions with numerical value response options. Free text responses were analysed using thematic analysis. *Results*: Overall there were 23 responses to the survey from participants across 19 different European countries, with a 95% country-level response rate. Serious concerns were expressed about GPs’ knowledge and understanding of ME/CFS, and, it was felt, about 60% of patients with ME/CFS went undiagnosed as a result. The vast majority of GPs were perceived to lack confidence in either diagnosing or managing the condition. Disbelief, and misleading illness attributions, were perceived to be widespread, and the unavailability of specialist centres to which GPs could refer patients and seek advice and support was frequently commented upon. There was widespread support for more training on ME/CFS at both undergraduate and postgraduate levels. *Conclusion*: The results of this survey are consistent with the existing scientific literature. ME/CFS experts report that lack of knowledge and understanding of ME/CFS among GPs is a major cause of missed and delayed diagnoses, which renders problematic attempts to determine the incidence and prevalence of the disease, and to measure its economic impact. It also contributes to the burden of disease through mismanagement in its early stages.

## 1. Introduction

Myalgic encephalomyelitis/chronic fatigue syndrome (ME/CFS) is a complex multi-system disorder that is characterised by a range of symptoms that can fluctuate in severity and change over time. These symptoms include post-exertional malaise, incapacitating fatigue that is not alleviated by rest, cognitive dysfunction, sleep disturbances, and muscle pain, while the condition can cause severe diminution in functioning and in quality of life [1].

It is estimated that there are around two million people with ME/CFS in the European Union and the United Kingdom combined, with an economic impact in the region of €40 billion per annum [2]. However, there are considerable difficulties in determining accurate prevalence and cost estimates, for a number of reasons. These include differences in case definitions, lack of empirical information in much of Europe about incidence and prevalence, natural variation between populations in, for example, the proportion of severely affected people, the heterogeneity of national economies and health care systems and, perhaps most importantly, the unwillingness of many doctors, particularly in primary care, either to recognise the condition as a genuine clinical entity or to diagnose it [2,3].

A recent literature review, covering studies from a wide variety of geographical locations world-wide over a 14-year period, found that between a third and a half of general practitioners (GPs) were unwilling to recognise or diagnose ME/CFS, that a similar proportion of patients were dissatisfied with the quality of primary care they had received, and that these proportions varied little over time [4]. In order to investigate this further, and to assess how knowledge and understanding of ME/CFS is perceived by experts in the condition across Europe, we conducted a survey of participants in the European ME/CFS Research Network (EUROMENE) project. EUROMENE was established to promote collaborative research on ME/CFS across Europe and is currently in receipt of EU funding from the COST Association (COST Action 15111) to support network activities. The aims of EUROMENE include reviewing the current state of the art and identifying gaps in knowledge about ME/CFS.

## 2. Materials and Methods

A survey questionnaire was sent to national representatives and members of the EUROMENE Core Group and Management Committee in September 2020—see Supplementary Materials. The questionnaire included a number of separate questions relating to: (i) the existence of GP patient lists and national guidance on treatment pathways; (ii) percentages of people with ME/CFS undiagnosed and presenting to a GP; (iii) percentages of GPs recognising, confident to diagnose, and confident to manage ME/CFS; (iv) percentages of patients diagnosed by GP, referred by GP to specialist care, and self-referring to specialist services; and (v) views on needs for teaching, training, reference literature, and referral centres. Responses to these questions were collated and summarized based on the numbers and percentages of respondents selecting each available option and these summary statistics were then used to generate a range of charts to clearly illustrate our main findings. Where options given in questions were in the form of percentages (e.g., 20–40%), weighted averages were calculated assuming the mid-points of the presented percentage ranges.

The questionnaire also included four questions seeking free text responses. The first such question sought purely factual information concerning the existence or otherwise of official guidance in respondents’ countries on the management of ME/CFS. The other three questions requested opinions on what constitutes specialist care in respondents’ countries, ways to increase knowledge and understanding among GPs, and other comments. The responses to these three questions were analysed using thematic analysis [5].

## 3. Results

### 3.1. Survey Question Responses

In total there were 23 responses received from EUROMENE members across 19 countries, namely: Austria, Belgium, Bulgaria, Denmark, Finland, France, Germany (3 responses), Greece, Ireland, Italy, Latvia, Netherlands, Norway, Poland, Romania, Serbia, Slovenia, Spain (3 responses), and the UK. With 20 member countries in EUROMENE, this represents a 95% country-level response rate. Where multiple responses were received from a country (namely Germany and Spain), we first examined the consistency of responses at a country level. Since there were some differences in responses for these countries for some questions, we chose to use data from all 23 responses in our analysis. Overall, however, our key results and findings do not alter significantly if we instead use a measure of the average response for these countries (results available on request).

In terms of the professional roles of respondents, 9 indicated that they were academics, 8 that they were medical consultants (e.g., neurology, internal medicine, infectious disease, psychology), 8 that they were GPs, 2 that they were retired, while 2 did not specify. Some respondents reported dual roles e.g., consultant and academic positions.

**Figure 1:**
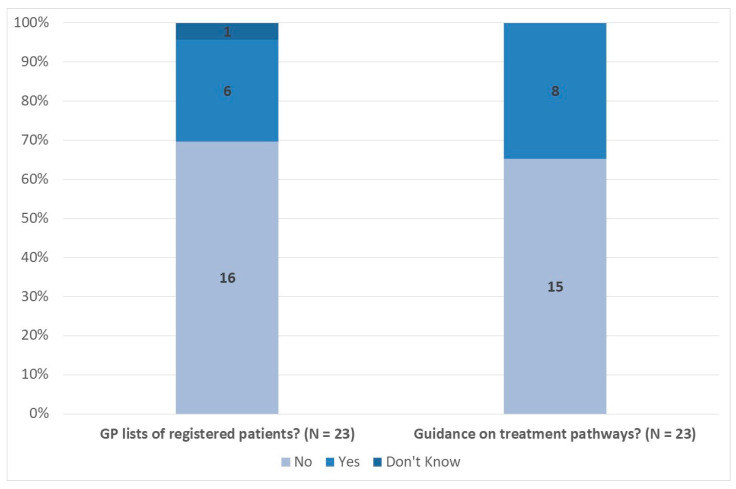
Existence of GP Patient Lists and National Guidance on Treatment Pathways (% Respondents). Note: Data labels indicate number of respondents per category. Source: EUROMENE Survey of Diagnosis and Management of ME/CFS in Primary Care in Europe, 2020.

To start, Figure 1 presents a summary of survey responses relating to the existence of GP patient lists and national guidance on treatment pathways. Overall 16 respondents (70%) reported there are no GP lists of registered patients in their country, while 15 (65%) reported no specific national guidance on treatment pathways.

**Figure 2:**
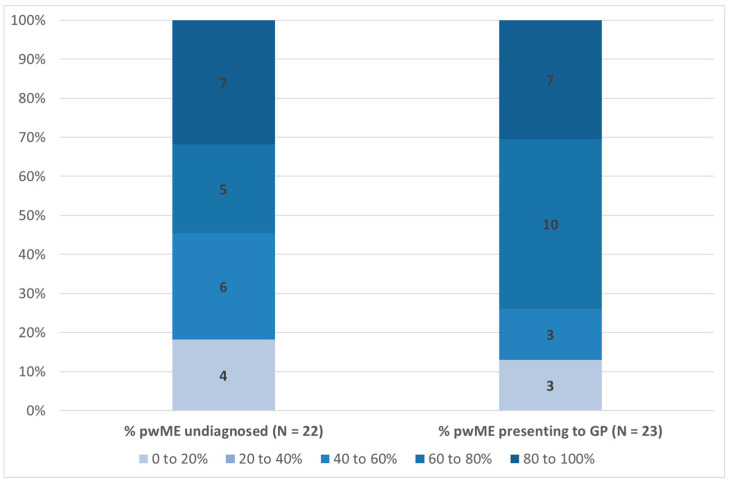
Percentage of People with ME/CFS Undiagnosed and Percentage Presenting to a GP (% Respondents). Note: Data labels indicate number of respondents per category. pwME denotes person with ME/CFS. Source: EUROMENE Survey of Diagnosis and Management of ME/CFS in Primary Care in Europe, 2020.

Figure 2 presents results relating to the percentage of people with ME/CFS that remain undiagnosed and the percentage that present to a GP. In relation to the former, there is considerable variation across respondents, with 4 respondents (18%) reporting between 0% and 20% remain undiagnosed, 6 (27%) that the proportion is 40–60%, 5 (23%) that it is 60% and 80%, and 7 (32%) that between 80% and 100% remain undiagnosed. It should be noted that one respondent did not answer this question. Taking a weighted average of all responses, and assuming a mid-point value for each option, gives an estimated average of 60% of people with ME/CFS remaining undiagnosed across our survey responses. Figure 2 also includes responses relating to the percentage of people with ME/CFS that present to a GP. Here 3 respondents (13%) reported that 0–20% do so, 3 (13%) that it is 20–40%, 10 (44%) that it is 60–80%, and 7 (30%) reported that between 80% and 100% of people with ME/CFS present to a GP. The weighted average is 66%, implying the majority present to a GP.

**Figure 3:**
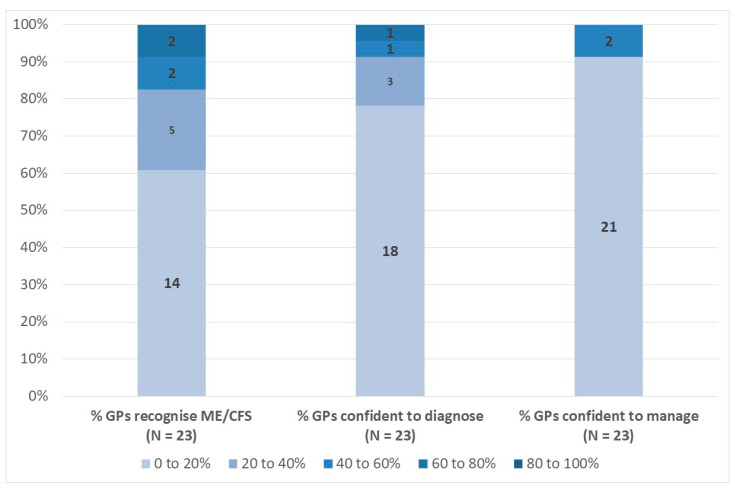
Percentage of GPs Recognising, Confident to Diagnose, and Confident to Manage ME/CFS (% Respondents). Note: Data labels indicate number of respondents per category. Source: EUROMENE Survey of Diagnosis and Management of ME/CFS in Primary Care in Europe, 2020.

Responses to the questions relating to GP recognition, confidence in diagnosing, and confidence in managing ME/CFS are presented in Figure 3. For GP recognition of the condition, 14 respondents (61%) chose the 0–20% option, while 5 (22%) reported 20–40%. Only a small number of respondents selected the other options and the weighted average estimate of the percentage of GPs recognising ME/CFS as a genuine clinical entity was 23%. Similar yet more pronounced patterns are evident for the diagnosis and management of ME/CFS. For example, 18 respondents (78%) reported that between 0% and 20% of GPs are confident of their ability to diagnose ME/CFS, with a weighted average of 17%. For management, 21 respondents (91%) reported that between 0% and 20% of GPs are confident of their ability to manage ME/CFS patients, with a weighted average of just 14%.

**Figure 4:**
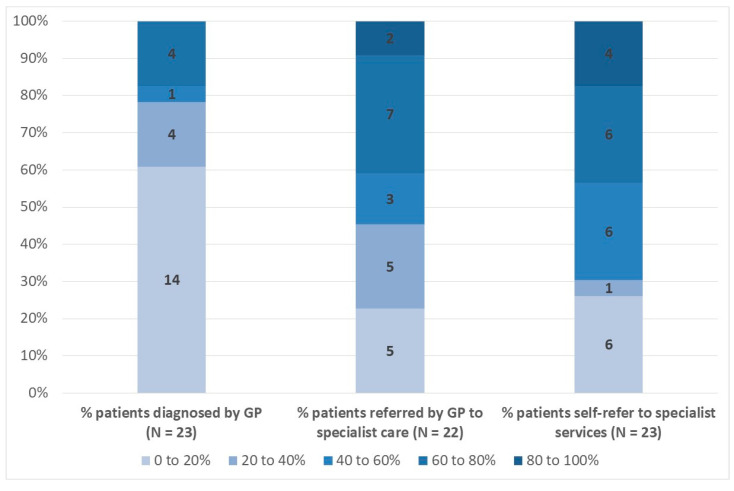
Percentage of Patients Diagnosed by GP, Referred by GP to Specialist Care, and Self-Referring to Specialist Services (% Respondents). Note: Data labels indicate number of respondents per category. Source: EUROMENE Survey of Diagnosis and Management of ME/CFS in Primary Care in Europe, 2020.

Figure 4 presents an overview of responses to the questions relating to the percentages of patients that are diagnosed by their GP, that are referred by their GP to specialist care, and that self-refer to specialist services. Overall, 14 respondents (61%) reported that between 0% and 20% of patients with ME/CFS who consult with their GP are in fact diagnosed by them, with 4 (18%) reporting the proportion to be 20–40% and 40–80%. The weighted average estimate from these respondents is 26%. There was more variation across responses to the question relating to GP referral to specialist care. For example, 5 respondents (23%) reported this to be 0–20% in their country, 5 (23%) that it is 20–40%, 3 (14%) that it is 40–60%, 7 (32%) that it is 60–80%, and 2 (9%) that 80–100% of patients that present to a GP are referred by the GP to specialist care. The weighted average response is 46%. Finally, Figure 4 also shows the responses relating to self-referral to specialist services. Again there is quite a lot of variation across respondents for this question, possibly due to variability in how this question was interpreted by ME/CFS specialists versus other specialists, and the weighted average percentage of patients self-referring is an estimated 51%.

**Figure 5:**
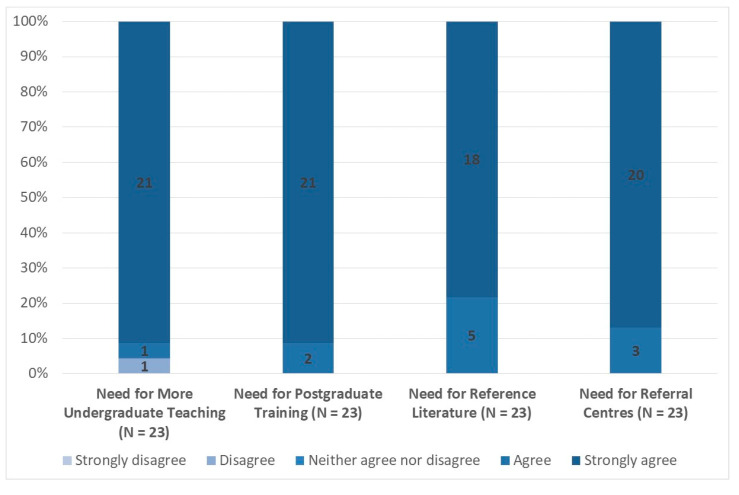
Views on Needs for Teaching, Training, Reference Literature, and Referral Centres (% Respondents). Note: Data labels indicate number of respondents per category. Source: EUROMENE Survey of Diagnosis and Management of ME/CFS in Primary Care in Europe, 2020.

Responses to the questions relating to the needs for teaching, training, reference literature, and referral centres are presented in Figure 5. Given the very high levels of consistency in responses across these responses, there is little need to discuss them in detail. Nonetheless, it is important to note that 21 respondents (91%) strongly agreed that there should be more teaching about ME/CFS in undergraduate medical curricula, while 21 respondents (91%) strongly agreed that postgraduate training about ME/CFS should be available for doctors and other healthcare professionals. For the latter question, the remaining survey respondents agreed with the statement. In addition, all respondents either strongly agreed or agreed that there is a need for succinct reference literature on ME/CFS for doctors and other healthcare professionals in primary care and that there is a need to ensure the existence of adequate secondary and tertiary referral centres for ME/CFS, from which primary care doctors could seek help and advice when necessary.

### 3.2. Analysis of Free Text Responses

#### 3.2.1. Official Guidance on ME/CFS

Respondents were asked to indicate whether official guidance on ME/CFS for healthcare professionals existed in their countries. Their free text responses indicated that such guidance was available and accessible, or under development, in Belgium, Germany, Italy, the Netherlands, Norway, Slovenia, Spain, the United Kingdom, and Finland. In Ireland, while there were no guidelines, clinicians tended to follow the UK NICE guidelines. The responses are summarised below.

Belgium has guidelines on diagnosis and clinical pathways from the government’s illness insurance programme. In Germany, there is a guideline on fatigue in general, which also covers ME/CFS, but it is said to be quite superficial and to lack understanding of the disease, and is therefore unhelpful. In Italy, a document was published in 2014 on behalf of AGENAS (the National Agency for Regional Health Services), which is part of the Health Ministry. It promotes a multidisciplinary approach to ME/CFS, with advice on aetiology, physiopathology, clinical features, diagnosis, and treatment. Practice in the Netherlands was reported to be based on American guidance from 2015. This likely refers to guidance published by the Institute of Medicine (IOM), now the National Academy of Medicine (NAM), entitled “Beyond Myalgic Encephalomyelitis/ Chronic Fatigue Syndrome: Redefining an Illness”, and summarised in a guide for healthcare providers [6]. Norway published guidance in 2014, and this was revised in 2015. It covers interdisciplinary investigation, diagnosis, treatment, rehabilitation and care, in various care locations (outpatients, inpatients, rehabilitation institutions, and self-management programmes) [7].

In Slovenia, EULAR recommendations for the management of fibromyalgia are followed [8]. In Spain, the public health system published a guide in 2019, but this guide was withdrawn, and patient associations and doctors who treat patients with ME/CFS have requested a complete review. It is also suggested that AQUAS 2017 is followed. AQUAS (Aggregated Quality Assurance for Systems) is an EU supported project promoting a holistic approach to safety, security, and performance in system development, medicine being one of the priority areas [9]. However, this appears rather remote from the diagnosis and management of patients with ME/CFS. In the United Kingdom, the NICE (National Institute for Health and Care Excellence) guidelines for ME/CFS are currently being revised [10]. Finland has official guidance in preparation. While it includes little on treatment pathways, there will be confirmation that pacing has a role in management, and that graded exercise therapy (GET) and cognitive behaviour therapy (CBT) will not be considered as effective treatment, which is very much in line with the current UK draft guideline from NICE.

#### 3.2.2. Specialist Care

Themes identified in the responses included the inadequacy of specialist care, the nature of the illness, specialties involved in care, multidisciplinary approaches, GP involvement, psychiatric involvement in care, the content of therapy, and the role of specialist centres. Emergent sub-themes regarding the nature of the illness included reporting of widespread disbelief in ME/CFS, as well as concern that it was regarded as a psychiatric, functional, or psychosomatic disease. On the content of therapy, emergent sub-themes were the use of CBT and GET, the involvement of rehabilitation institutions, and the role of laboratory investigations and psychological examination. A detailed examination of sub-themes is summarised below.

The central role of dedicated specialists was reported from Latvia and of specialised centres from Spain. There was one such dedicated specialist cluster in Latvia, and certain specific centres in Spain, with a role in diagnosis and management. Widespread disbelief in the existence of ME/CFS was reported as a factor limiting provision of specialist care in Greece. There was reported to be no specialist care available in Austria, Denmark, the Netherlands or Rumania, and little available in France, Germany, Ireland, or Poland. However, since specialist was not defined explicitly in the survey, it is possible that respondents may be referring to a lack of ME/CFS specialists, rather than saying that people with ME/CFS don’t have access to internal medicine and rehabilitation medicine specialists, neurologists, etc. Collaboration with GPs was seen as important in specialist care delivery in Belgium and Slovenia, but seen as problematic in Italy. In Ireland and the UK, GP referral is important as the gateway to specialist care, though this may be verging on non-existent or involve prolonged delays. The two specialist centres in Germany are involved in teaching GPs.

Psychiatric involvement in specialist care was widespread. ME/CFS was perceived to be seen as a functional or psychiatric disease in Austria and Finland, and in Belgium psychiatrists were involved in care. In Italy and the UK, multidisciplinary care involved psychiatrists and psychologists, and psychologists were also involved in care in Serbia. A variety of clinical specialties are involved in the specialist care of people with ME/CFS, though are few in number in most countries. Neurologists are most frequently mentioned, in France, Greece, Italy, Latvia, Slovenia, and Spain. In the UK, various specialties are involved, which would include neurology. Internal medicine specialists are mentioned as being involved in Belgium, France, Italy, Latvia, and Spain. In France and Italy, the involvement of immunologists was mentioned. In Italy and Latvia, the involvement of infectious disease specialists was mentioned. Physical medicine involvement was reported from Italy, rehabilitation medicine from Norway, exercise physiology from Poland, and rheumatology from Slovenia and Spain. There was little information volunteered regarding the content of therapy delivered in specialist care, though CBT and GET were reported from Belgium. The involvement of rehabilitation institutions was reported from Norway, and the role of laboratory investigations and psychological examination from Serbia. A multidisciplinary approach was reported as important in specialist care in Italy, Norway, Slovenia and the UK.

#### 3.2.3. Increasing GP Knowledge and Understanding

Respondents were asked if they had any other suggestions as to possible ways to increase the knowledge and understanding of ME/CFS among primary care doctors and/or other healthcare professionals in primary care. Emergent themes from the responses were the inadequacy of current approaches, the need for top-down action to improve the situation, the importance of centres of excellence, the question of who needed training, the content of curricula, and communications strategies. The inadequacy of current approaches was reported from Austria, where it was stated that few healthcare professionals were involved in the care of people with ME/CFS and interest in the topic was largely down to chance. In Finland similarly, the approach was seen as inadequate because it reinforced the belief that ME/CFS was a functional disorder.

Suggested top-down approaches to improve the situation included the suggestion from Austria that such an approach was needed to establish specialist centres, while from the Netherlands there was seen to be a need for consensus and advice in order to establish such centres. From Germany, it was suggested that standard operating procedures were needed Europe-wide or even world-wide. A similar approach was suggested from Latvia through the development of clinical algorithms, while from Poland financial support for diagnosis and therapy was seen as a priority. In France also, the development of specialist centres was seen as a priority, and in Germany such a centre provided information and education for physicians and patients.

The need for education and training about ME/CFS in undergraduate curricula was raised in Greece, and for Belgium it was suggested that curricula should include the neurophysiology of chronic pain and fatigue. The question of who required training was addressed from Germany, and it was suggested that not only GPs but also neurologists, psychiatrists, cardiologists, endocrinologists, rheumatologists, oncologists, infectious disease specialists, and other specialists required training. Suggestions for possible communications strategies included video-talks, flyers in all general practice premises, health ‘passports’ with basic information on ME/CFS, local and national meetings involving patients’ associations, webinars, on-line short courses and Massive Open Online Courses (MOOCs), and information for the general public on overlapping syndromes like fibromyalgia.

#### 3.2.4. Final Comments

Final comments were ventured from six countries. In Austria, the lack of a systematic plan for ME/CFS was stressed, as was the difficulty patients experienced in medical care and also as regards social insurance through dismissal of their illness. From Denmark it was pointed out that the history of ME/CFS could aid understanding of Covid and its sequelae. In Finland, it was felt that, despite current unhelpful attitudes and official opposition, the healthcare system was on the threshold of a change in attitudes, and patience, more research, and effective treatments were needed to bring this about. However, in Slovenia there was less optimism about the future. Finally, the French contribution was philosophical, quoting William 1st of Orange-Nassau: “*Il n’est pas nécessaire d’espérer pour entreprendre, ni de réussir pour persévérer*” (“*It is not necessary to hope to embark on a course of action, nor to succeed in order to persevere*”).

## 4. Discussion

This survey has identified serious concerns among academic and clinical experts on ME/CFS about the state of knowledge and understanding among primary care physicians across a large number of European countries. Overall, based on respondents’ experience, it was estimated that around 60% of patients went undiagnosed, and while about two-thirds of patients were estimated to have consulted their GPs and around a quarter were diagnosed by them, the vast majority of GPs were perceived to lack confidence in either diagnosing or managing the condition. About half of patients diagnosed by their GPs were not referred for specialist care, which is not surprising since many countries lack specialist care facilities to which patients could be referred. Disbelief on the part of doctors, or misleading illness attributions, are seen as major factors impeding patients’ access to care. There was almost unanimous support for proposed solutions to the problem, in particular including more teaching about ME/CFS in undergraduate medical curricula and in postgraduate training programmes, as well as providing accessible reference material in primary care, and making advice and support available from specialist centres. In order for this to happen, though, there was a perceived need to develop specialist ME/CFS centres in the majority of countries where they currently do not exist at all or only on a very small scale. To achieve this, there was a prior need to develop consensus about their role and their modus operandi, and to identify the necessary resources.

The strength of this study is that, for the first time, it has been possible to conduct a Europe-wide study to elicit the views of ME/CFS experts on the problem of apparent lack of knowledge and understanding of ME/CFS among primary care physicians. Its weakness is that we were unable to survey GPs directly in the participating countries, though there were 8 GP respondents in our sample, and further research is needed to remedy this deficiency. A further weakness is that since our respondents work in the ME/CFS area there may be some bias in responses, particularly given that the survey questions relate mainly to opinions. In addition, it is important to acknowledge that depending on the specific question, respondents may have varied first-hand knowledge/experience on which to base their responses, given that they have different backgrounds and roles with respect to ME/CFS.

Surveys of GPs on this topic have been confined to Ireland [11] and the UK [12,13,14], and it has been assumed by inference that similar conclusions could be applied more widely across European countries. In all four studies, large minorities of GPs did not accept the existence of ME/CFS as a genuine clinical entity. In the Irish study published in 1997 [11], this proportion was 42% of respondents. The UK studies were from in Scotland in 2005 [12], South Wales in 1991 [13] and South-West England in 2005 [14], and the proportions of respondents not accepting the genuineness of ME/CFS as a diagnosis were 29%, 46% and 28% respectively. We recently carried out a literature review, looking more widely at the question and took into consideration qualitative reports, patient surveys and grey literature, which covered a wider time span and a more extensive geographical area [4]. In particular, our review covered the period from 1946 until 20 August 2020 and included studies from more than 10 countries. It concluded that between a third and a half of GPs lack confidence in diagnosing or managing ME/CFS, with many disputing its existence, while a similar proportion of ME/CFS patients express dissatisfaction with the primary medical care they have received. This has implied an important role for ME/CFS patient associations in the provision of assistance to patients and the dissemination of knowledge about the disease [15]. The findings of our survey are thus broadly in line with the pre-existing scientific literature.

The problem of deficiencies in GP knowledge and understanding of ME/CFS, and an apparent high level of missed and delayed diagnoses, not only impedes attempts to quantify both the prevalence and incidence of the disease, as well as the economic impact of ME/CFS, but is also likely to increase the economic burden attributable to it, since mismanagement of the early stages of the illness is a known risk factor for severe, prolonged disease [16]. Further work to be undertaken should therefore focus, not only on surveys designed to quantify the scale of the problem in different European countries, but also on developing initiatives to improve GP knowledge and understanding, and to facilitate the diagnostic process in primary care.

## 5. Conclusions

A group of academic and clinical experts on ME/CFS, from nineteen European countries, were strongly of the opinion that lack of knowledge and understanding of the illness among primary care physicians, including disbelief in the very existence of the condition as a genuine clinical entity, was very widespread. As a result, they believed a high proportion had gone undiagnosed. There were seen to be inadequacies in both undergraduate and postgraduate teaching about the illness, and a lack of sources of advice and information for primary care. To address this, ME/CFS specialist centres for referral, support, and advice were needed, but in a majority of countries these either did not exist, or where present, existed only in certain restricted geographical locations. Further research is needed to survey GPs’ attitudes in participating countries, and to develop programmes to inform and support healthcare professionals in the primary care sector.

## Data Availability

De-identified responses to the survey questions are available on request from the corresponding author.

## Supplementary Materials

The following are available online at https://www.mdpi.com/1648-9144/57/3/208/s1, Questionnaire: Survey of Diagnosis and Management of ME/CFS in Primary Care in Europe.
