# Supplementary material for: Perceptions of European ME/CFS Experts Concerning Knowledge and Understanding of ME/CFS among Primary Care Physicians in Europe: A Report from the European ME/CFS Research Network (EUROMENE)
Source: Medicina (Kaunas). 2021 Feb 26;57(3):208. doi: 10.3390/medicina57030208 (PMC7996783; doi:10.3390/medicina57030208)
Supplement: Supplementary file 1 [file medicina-57-00208-s001.pdf]

SUPPLEMENTARY MATERIALS: The Questionnaire to EUROMENE Participants

COST Action 15111 – EUROMENE

Working Group 3 - Socioeconomics

Survey of Diagnosis and Management of ME/CFS in Primary Care in Europe

Name (optional)
Institution (optional)
Your professional role (optional)
Country (residence and/or place of work)

In your country:-

	Yes	No	Don't know
Do GPs have lists of registered patients?			
Is there specific national guidance on treatment pathways (as for example in England via NICE)?			
If 'yes', please give brief details:--			
What constitutes "specialist care" for ME/CFS in your country? Please give brief details:-			

The following questions concern your impression of the management of ME/CFS in primary care. We are not asking you to conduct extensive research, but rather to answer the questions on the basis of your existing understanding of the current position in primary care.

	0 < 20%	20 < 40%	40 < 60%	60 < 80%	80 < 100%
What percentage of people with ME/CFS remain undiagnosed?					
What percentage of people with ME/CFS present to a GP?					
What percentage of GPs recognise ME/CFS as a genuine clinical entity?					

What percentage of GPs are confident of their ability to diagnose ME/CFS?					
What percentage of GPs are confident of their ability to manage patients with ME/CFS?					
What proportion of patients with ME/CFS who consult their GPs are in fact diagnosed by them?					
What proportion of patients with ME/CFS who present to a GP are referred by the GP to specialist care?					
What percentage of patients with ME/CFS self-refer to specialist services?					

Are then any reports in your country relevant to primary care ascertainment or management of ME/CFS in your country? If so, please attach link(s) here:-

For the remaining questions, please indicate the extent to which you agree or disagree with the following statements:-

	Strongly agree	Agree	Neither agree nor disagree	Disagree	Strongly disagree
There should be more teaching about ME/CFS in undergraduate medical curricula					
Postgraduate training about ME/CFS should be available for doctors and other healthcare professionals					
There is a need for succinct reference literature on ME/CFS for doctors and other healthcare professionals in primary care					
There is a need to ensure the existence of adequate secondary and tertiary referral centres for ME/CFS, from which primary care doctors could seek help and advice when necessary					

Do you have any other suggestions as to possible ways to increase the knowledge and understanding of ME/CFS among primary care doctors and/or other healthcare professionals in primary care?

Are there any other comments you wish to make?

Thank you very much for taking part in this survey.
